# Differentiation of benign and malignant parotid gland tumors based on the fusion of radiomics and deep learning features on ultrasound images

**DOI:** 10.3389/fonc.2024.1384105

**Published:** 2024-05-13

**Authors:** Yi Wang, Jiening Gao, Zhaolin Yin, Yue Wen, Meng Sun, Ruoling Han

**Affiliations:** Department of Ultrasound, The Fourth Hospital of Hebei Medical University, Shijiazhuang, Hebei, China

**Keywords:** radiomics, deep learning, feature fusion, parotid gland tumors, nomogram, ultrasound

## Abstract

**Objective:**

The pathological classification and imaging manifestation of parotid gland tumors are complex, while accurate preoperative identification plays a crucial role in clinical management and prognosis assessment. This study aims to construct and compare the performance of clinical models, traditional radiomics models, deep learning (DL) models, and deep learning radiomics (DLR) models based on ultrasound (US) images in differentiating between benign parotid gland tumors (BPGTs) and malignant parotid gland tumors (MPGTs).

**Methods:**

Retrospective analysis was conducted on 526 patients with confirmed PGTs after surgery, who were randomly divided into a training set and a testing set in the ratio of 7:3. Traditional radiomics and three DL models (DenseNet121, VGG19, ResNet50) were employed to extract handcrafted radiomics (HCR) features and DL features followed by feature fusion. Seven machine learning classifiers including logistic regression (LR), support vector machine (SVM), RandomForest, ExtraTrees, XGBoost, LightGBM and multi-layer perceptron (MLP) were combined to construct predictive models. The most optimal model was integrated with clinical and US features to develop a nomogram. Receiver operating characteristic (ROC) curve was employed for assessing performance of various models while the clinical utility was assessed by decision curve analysis (DCA).

**Results:**

The DLR model based on ExtraTrees demonstrated superior performance with AUC values of 0.943 (95% CI: 0.918-0.969) and 0.916 (95% CI: 0.861-0.971) for the training and testing set, respectively. The combined model DLR nomogram (DLRN) further enhanced the performance, resulting in AUC values of 0.960 (95% CI: 0.940- 0.979) and 0.934 (95% CI: 0.876-0.991) for the training and testing sets, respectively. DCA analysis indicated that DLRN provided greater clinical benefits compared to other models.

**Conclusion:**

DLRN based on US images shows exceptional performance in distinguishing BPGTs and MPGTs, providing more reliable information for personalized diagnosis and treatment plans in clinical practice.

## 1 Introduction

The parotid gland is a vital exocrine organ and the primary site for salivary gland tumors. Parotid gland tumors (PGTs) account for approximately 3-12% of head and neck neoplasms, with 80% of all salivary gland tumors occurring in this location (1, 2). The majority of these tumors are benign, comprising around 75% to 80%,with pleomorphic adenomas (PA) and Warthin tumors being the most common types, followed by basal cell adenomas (BCA). Mucoepidermoid carcinoma (MEC) is the most frequent malignant parotid gland tumor (MPGTs), followed by adenoid cystic carcinoma (ACC) and acinar cell carcinoma (3, 4). The pathological subtypes of PGTs are complex. Accurate discrimination between benign and malignant PGTs is crucial for clinical management and prognosis assessment. For most benign parotid gland tumors (BPGTs), partial gland or simple tumor resection suffices (5). However, MPGTs often require more aggressive interventions such as total parotidectomy along with potential lymph node dissection, complemented by radiotherapy if deemed necessary (6).

Preoperative auxiliary diagnosis of PGTs primarily involves two methods: fine-needle aspiration cytology (FNAC) and imaging examination. FNAC is currently widely utilized as an adjunctive diagnostic tool, exhibiting an accuracy rate ranging from 85% to 97% in distinguishing between BPGTs and MPGTs (7). However, due to the limited sample size, it may not fully represent the overall characteristics of the tumor, leading to inconclusive diagnoses (8). Furthermore, FNAC is an invasive procedure that carries risks of tumor cell implantation metastasis and inducing parotitis (9). Currently employed imaging techniques for parotid examination include ultrasound (US), computed tomography (CT),and magnetic resonance imaging (MRI). CT can effectively illustrate the relationship between the tumor and surrounding tissue structures. MRI offers high soft tissue resolution enabling assessment of nerve invasion by tumors. Nevertheless, their clinical application is restricted by ionizing radiation exposure, high costs, and various contraindications (10, 11). In comparison, US possesses non-invasive features with real-time capability at a lower cost. It provides comprehensive information regarding the location, size, shape, margin, and blood supply of tumors; hence, it is considered as the preferred preoperative imaging method for evaluating PGTs (12). Nonetheless, the US features of PGTs partially overlap, and interpretation of US findings may vary depending on operator experience, resulting in discrepancies in diagnostic outcomes (13).

Radiomics is a field emerged from the convergence of artificial intelligence (AI) and medical imaging. It enables the extraction of potential features from medical images that are imperceptible to the human eye in a high-throughput manner, which can be transformed into visual data for quantitative analysis (14). By utilizing machine learning models, radiomics facilitates non-invasive assessment of various biological behaviors associated with tumors, making it widely applicable in early diagnosis, prognosis prediction, and treatment evaluation (15–17). While several scholars have conducted radiomics research on PGTs using CT and MRI images (18–20), there is limited literature based on US images (21).

In recent years, the rapid development of AI has led to the widespread application of deep learning (DL) in various medical fields. Among different types of DL architectures, convolutional neural networks (CNNs) have emerged as the most commonly used approach (22). Compared to traditional radiomics, DL neural networks with their multi-layer structure can automatically learn semantic and spatial features from hidden layers, enabling end-to-end mapping from input to output. This capability has shown promise in improving tumor classification performance (23–25). Yu et al. (26) developed multiple DL models based on multi-center CT images to assist in diagnosing BPGTs and MPGTs, and it was found that MobileNet V3 exhibited the best predictive performance. When compared to the traditional radiomic SVM model, MobileNet V3 demonstrated a significant increase in sensitivity by 0.111 and 0.207 for internal and external test sets respectively (P < 0.05). The utilization of these models resulted in notable improvements in clinical benefits and overall efficiency for less experienced radiologists.

The traditional radiomics methods have complex workflows and primarily rely on manually defined features, which may not fully capture the inherent heterogeneity within lesions. Although DL has the potential to automatically learn more comprehensive features, its algorithms are abstract and less interpretable. While radiomics and DL features have their own distinct advantages and limitations, their integration offers complementary information, making it a prominent research direction in recent years. To our knowledge, there is currently no existing research that utilizes fusion models of radiomics and DL features for characterizing the differentiation of BPGTs and MPGTs including US, CT, and MRI. We hypothesize that fused features can offer additional valuable information to enhance the efficacy of US radiomics in distinguishing between BPGTs and MPGTs. In this study, we compared the diagnostic performance of multiple radiomics classifier models with various DL models. Additionally, we developed a feature fusion model and integrated clinical and US features to construct a nomogram, aiming to enhance the visual classification of preoperative diagnosis for PGTs and facilitate personalized precision diagnosis and treatment for patients.

## 2 Materials and methods

### 2.1 Patients

The present study has obtained approval from the hospital ethics committee (protocol code 2024KS002). Given its retrospective nature, patient informed consent was waived.

**Figure 1:**
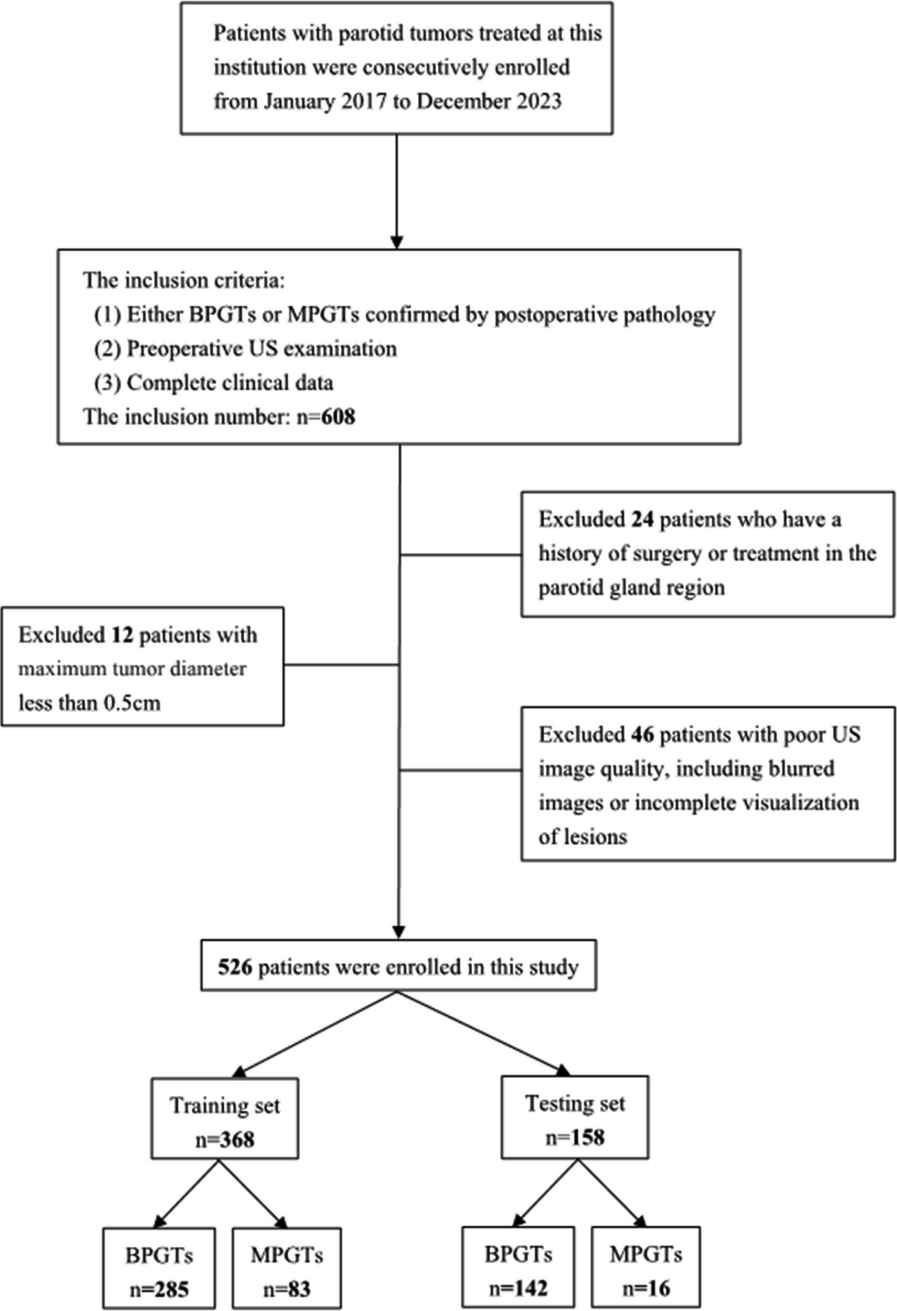
The patient recruitment process and distribution in the training and testing sets.

A retrospective analysis was conducted on US images obtained from January 2017 to December 2023, involving a consecutive cohort of 608 patients with PGTs who received treatment at our hospital. The inclusion criteria consisted of: (1) either BPGTs or MPGTs confirmed by postoperative pathology, (2) preoperative US examination, and (3) complete clinical data. Exclusion criteria included: (1) previous history of surgery or treatment in the parotid gland region, (2) maximum tumor diameter less than 0.5cm,and (3) poor image quality, including blurred images or incomplete visualization of lesions. In cases with multiple lesions, the largest or most representative malignant lesion was selected for analysis. Detailed recruitment methods can be found in **Figure 1**. Relevant clinical information including age, gender, smoking and drinking history, along with postoperative pathological results were retrieved from the Electronic Health Records (EHR) system.

**Figure 2:**
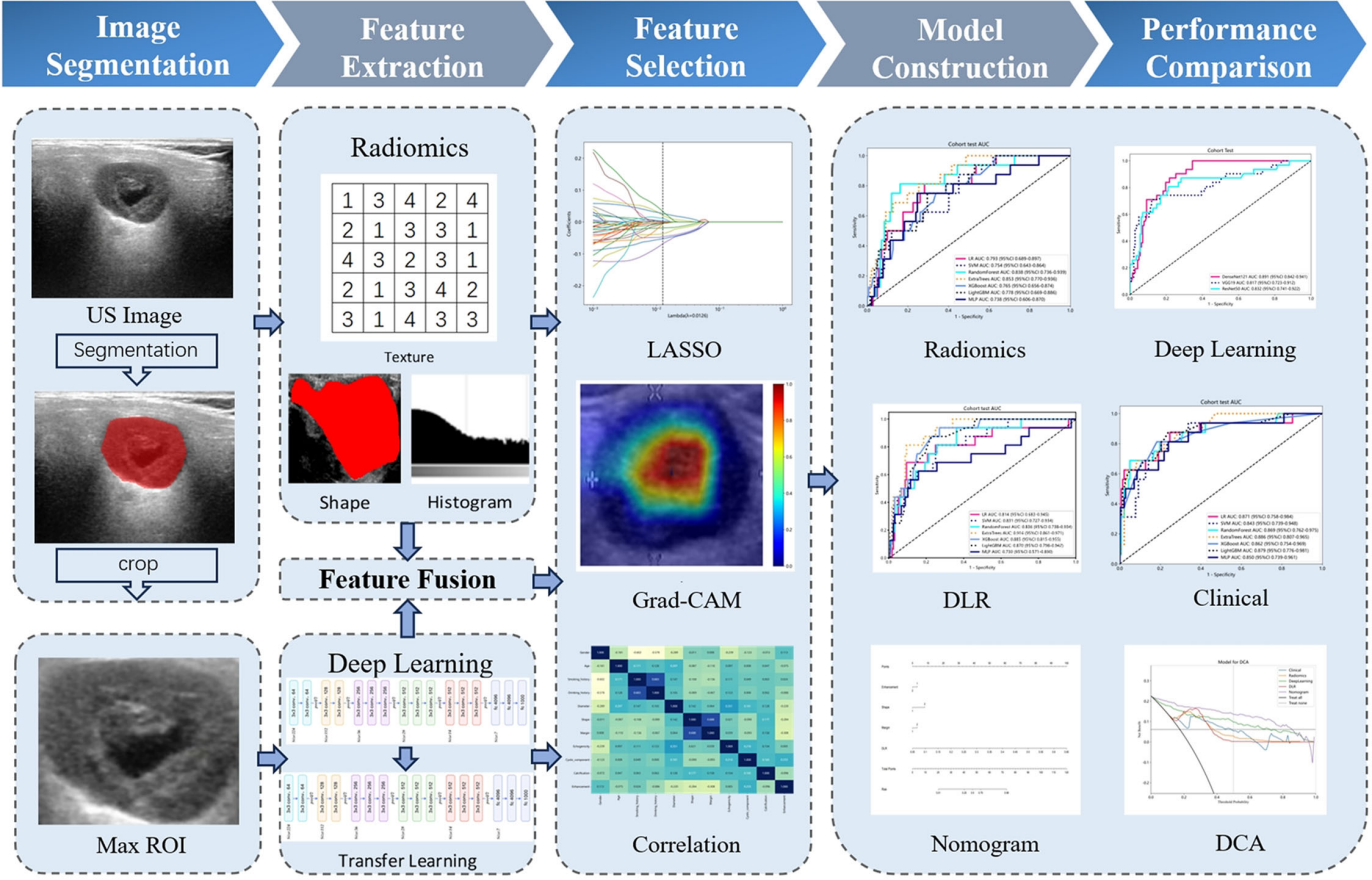
The overall workflow of this study.

The study enrolled a total of 526 patients, including 427 cases of BPGTs and 99 cases of MPGTs. These patients were randomly allocated to a training and testing set in a ratio of 7:3. The study design and workflow are illustrated in **Figure 2**.

### 2.2 Image acquisition and analysis

Preoperative US examination of the parotid gland region was performed using iU22 (PHILIPS), EPIQ7 (PHILIPS),S2000 (SIEMENS), and ACUSON Sequoia (SIEMENS) ultrasound diagnostic devices, equipped with corresponding high-frequency linear array probes. Two-dimensional US images of PGTs were acquired from the Picture Archiving and Communication System (PACS), capturing essential characteristics including maximum diameter, shape (regular/irregular), margin (well/poorly-defined),echogenicity (homogeneous/hetero-geneous), cystic component (absent/present), calcification (absent/present), and posterior acoustic enhancement (absent/present). The analysis of US images was independently conducted in a blinded manner by two experienced ultrasound physicians A and B (with over 5 years and 10 years of experience in superficial organ diagnosis respectively) without access to clinical information or pathological results. In case of discrepancies, consensus was reached through discussion.

### 2.3 Image segmentation

The ITK-SNAP software (version 3.8.0) was utilized for manual delineation of the region of interest (ROI) along the tumor periphery on images displaying the maximum lesion diameter. Initially, ultrasound physician A performed the ROI delineation, and subsequently, a subset of 100 patients were randomly selected after a two-week interval for independent ROI delineation by both ultrasound physicians A and B, aiming to assess the selected features with high reproducibility and robustness in terms of intra-observer and inter-observer agreement.

### 2.4 HCR feature extraction

Handcrafted radiomic (HCR) feature extraction was performed with the Pyradiomics (version 3.0.1), adhering to the Imaging Biomarker Standardization Initiative (IBSI) guidelines. The documentation for this program can be accessed at https://pyradiomics.readthedocs.io. HCR features are classified into three primary groups: (1) Geometry, (2) Intensity, (3) Texture. Geometry features are designed to characterize the spatial structure and contour of lesion; Intensity features analyze voxel intensity-related information using first-order statistical methods; and Texture features capture subtle variations in lesions through more intricate second- and higher-order analyses. Various techniques were utilized to extract texture features, including gray-level co-occurrence matrix (GLCM), gray-level dependence matrix (GLDM),gray-level run length matrix (GLRLM),gray-level size zone matrix (GLSZM),and neighborhood gray-tone difference matrix (NGTDM).

### 2.5 DL feature extraction

In order to ascertain the most suitable algorithm for our specific research requirements, we explored the performance of prominent networks including DenseNet121, VGG19, and ResNet50. To improve the generalization capability across diverse datasets, transfer learning was implemented by initializing the models with pre-trained weights from the ImageNet database and fine-tuning the learning rate using the cosine decay learning rate strategy. Further details regarding the specific definition and methodology can be found in **Supplementary Material 1**.

Prior to training, the input images underwent cropping and Z-score normalization, retaining only the minimum bounding rectangle that encompasses the ROI. This simplified complexity and reduces background noise in algorithmic analysis. During training, we employed real-time data augmentation techniques such as random cropping, horizontal flipping, and vertical flipping. For testing set images, only normalization was performed during processing.

The classification performance of three DL models was compared, and DL features were extracted from the penultimate layer (average pooling layer) of the most effective model for subsequent analysis.

### 2.6 Feature selection and fusion

For HCR features, the initial step involves calculating the intraclass correlation coefficient (ICC) between HCR features and retaining those with an ICC value ≥ 0.85, indicating a high level of stability. Subsequently, feature standardization is performed using Z-scores, complemented by intergroup comparisons based on t-test. Features exhibiting p-values < 0.05 were selected for further analysis. Furthermore, we examined repeatable features using Pearson’s correlation coefficient and opted to retain only one feature in cases where the correlation between feature pairs exceeded 0.9. To reduce redundancy further, a greedy recursive deletion strategy is employed for feature filtering. Finally, least absolute shrinkage and selection operator (LASSO) regression with cross-validation utilizing a minimum criterion of 10 folds is applied to adjust the penalty parameter (λ), aiming to identify HCR features among non-zero coefficients that possess superior predictive value.

For DL features, we applied principal component analysis (PCA) to reduce the dimensionality of these transfer learning features from 50,176 to 512, in order to enhance the model’s generalization ability and mitigate the risks of overfitting.

In the stage of feature fusion, we employed a pre-fusion algorithm that integrated HCR features with DL features to form a comprehensive feature set. Subsequently, we followed the similar process as that for HCR features for fusion feature selection.

### 2.7 Model construction and validation

HCR features and fused features obtained through feature selection are combined with several machine learning classifiers to construct traditional radiomics models and deep learning radiomics (DLR) models for discriminating BPGTs and MPGTs. Seven mainstream classifiers, including linear models (logistic regression (LR), support vector machine (SVM)),tree-based models (RandomForest, ExtraTrees, XGBoost, LightGBM), as well as a deep learning-based multi-layer perceptron (MLP) model were selected. For model hyperparameter tuning, we applied 5-fold cross-validation on the training set and utilized the Gridsearch algorithm. The model parameters that exhibited superior median performance were chosen for final model training.

Through a comprehensive analysis of relevant clinical data and US characteristics, we conducted univariate r followed by multivariate logistic regression analysis to identify significant features for constructing clinical models. Furthermore, these selected features were integrated with the most optimal predictive machine learning model to develop a nomogram.

The receiver operating characteristic (ROC) curve was employed for assessing the diagnostic performance of various models, while the Delong test was utilized to compare the area under the curves (AUC) of each model. Calibration curves and Hosmer-Lemeshow (HL) analysis were plotted to evaluate the concordance between predicted probabilities and actual outcomes. Decision curve analysis (DCA) was applied to assess the clinical utility of these models.

### 2.8 Statistical analysis

The analyses were performed using Python (version 3.7.12) and statsmodels (version 0.13.2). The development of our machine learning models utilized the scikit-learn (version 1.0.2) interface. DL training was conducted on an NVIDIA 4090 GPU, with MONAI 0.8.1 and PyTorch 1.8.1 frameworks.

For quantitative data, normality and homogeneity of variance tests were conducted. If the data followed a normal distribution, it was represented as mean ± standard deviation and an independent samples t-test was used for comparison. If the data did not follow a normal distribution, median and interquartile range (IQR) were used for representation, and a non-parametric Mann-Whitney U test was employed for comparison. For categorical data, a chi-square test was utilized for comparison. A significance level of P<0.05 indicated statistical significance.

## 3 Results

### 3.1 Clinical and US characteristics

Ultimately, a total of 526 patients were enrolled in the study, including 283 males and 243 females, with ages ranging from 12 to 87 years (mean age: 51.73 ± 15.17 years). Among the cohort of BPGTs (n=427), PA was the most prevalent subtype (207 cases; accounting for 48.48%), followed by Warthin tumor (133 cases; accounting for 31.15%). Of the MPGTs (n=99),MEC exhibited the highest proportion (28 cases; accounting for 28.28%). The distribution of tumors is presented in **Table 1**.

**Table 1:** Distribution of tumors confirmed by histologic results in the whole cohort.

Benign tumors	Number	Malignant tumors	Number
Pleomorphic adenoma	207	Mucoepidermoid carcinoma	28
Warthin tumor	133	Adenoid cystic carcinoma	17
Basal cell adenoma	40	Salivary duct carcinoma	15
Myoepithelioma	36	Acinic cell carcinoma	13
Others	11	Others	26

The baseline characteristics of the training and testing sets were compared in **Table 2**, and no statistically significant differences (P>0.05) were observed between the clinical and US characteristics of the two groups, ensuring an unbiased data partition. Extensive univariate and multivariate analyses were conducted on the baseline characteristics BPGTs and MPGTs to determine odds ratios (ORs) for each feature along with their corresponding p-values (**Supplementary Table 1**). Univariate analysis revealed significant differences (P < 0.05) between the two groups regarding smoking history, maximum diameter, shape, margin, calcification, and posterior acoustic enhancement. Multivariate analysis identified only irregular shape (OR=1.257), poorly-defined margin (OR=1.323),and absence of posterior acoustic enhancement (OR=0.807) as independent risk factors for MPGTs.

**Table 2:** Baseline clinical and US characteristics of patients in training and testing sets.

Clinical and US characteristics	All (n=526)	Training set (n=368)	Testing set (n=158)	*P*
Age(year)	51.73 ± 15.17	51.74 ± 15.48	51.70 ± 14.47	0.866
Maximum diameter(cm)	2.69 ± 0.98	2.69 ± 0.98	2.67 ± 0.97	0.8
Gender				0.923
Male	283(53.80)	199(54.08)	84(53.16)	
Female	243(46.20)	169(45.92)	74(46.84)	
Smoking history				1.0
Absent	356(67.68)	249(67.66)	107(67.72)	
Present	170(32.32)	119(32.34)	51(32.28)	
Drinking history				0.844
Absent	371(70.53)	261(70.92)	110(69.62)	
Present	155(29.47)	107(29.08)	48(30.38)	
Shape				0.111
Regular	376(71.48)	255(69.29)	121(76.58)	
Irregular	150(28.52)	113(30.71)	37(23.42)	
Margin				0.176
Well-defined	373(70.91)	254(69.02)	119(75.32)	
Poorly-defined	153(29.09)	114(30.98)	39(24.68)	
Echogenicity				0.332
Homogeneous	111(21.10)	73(19.84)	38(24.05)	
Heterogeneous	415(78.90)	295(80.16)	120(75.95)	
Cystic component				1.0
Absent	413(78.52)	289(78.53)	124(78.48)	
Present	113(21.48)	79(21.47)	34(21.52)	
Calcification				0.264
Absent	439(83.46)	312(84.78)	127(80.38)	
Present	87(16.54)	56(15.22)	31(19.62)	
Posterior acoustic enhancement				0.385
Absent	121(23.00)	89(24.18)	32(20.25)	
Present	405(77.00)	279(75.82)	126(79.75)	

Numbers in parentheses are percentages.

We performed numerical mapping on these features and subsequently modeled them by machine learning algorithms. The diagnostic performance of various clinical models was compared in **Table 3** and **Supplementary Figure 1**. Among all the models, ExtraTrees exhibited superior performance in the test set with an AUC of 0.886 (95% CI: 0.807 - 0.965).

**Table 3:** Performance comparison of different clinical models.

Model	AUC	95% CI	Accuracy	Sensitivity	Specificity	PPV	NPV
LR-training	0.918	0.886 - 0.949	0.804	0.964	0.758	0.537	0.986
SVM-training	0.952	0.929 - 0.975	0.902	0.904	0.902	0.728	0.970
RandomForest-training	0.908	0.875 - 0.940	0.821	0.843	0.814	0.569	0.947
ExtraTrees-training	0.903	0.868 - 0.937	0.826	0.843	0.821	0.579	0.947
XGBoost-training	0.897	0.860 - 0.934	0.829	0.843	0.825	0.583	0.948
LightGBM-training	0.916	0.885 - 0.947	0.851	0.819	0.860	0.630	0.942
MLP-training	0.941	0.917 - 0.965	0.851	0.940	0.825	0.609	0.979
LR-testing	0.871	0.758 - 0.984	0.759	0.812	0.754	0.271	0.973
SVM-testing	0.843	0.739 - 0.948	0.684	0.875	0.662	0.226	0.979
RandomForest-testing	0.869	0.762 - 0.975	0.918	0.625	0.951	0.588	0.957
ExtraTrees-testing	0.886	0.807 - 0.965	0.892	0.625	0.923	0.476	0.956
XGBoost-testing	0.862	0.754 - 0.969	0.848	0.687	0.866	0.367	0.961
LightGBM-testing	0.879	0.776 - 0.981	0.791	0.812	0.789	0.302	0.974
MLP-testing	0.850	0.739 - 0.961	0.759	0.750	0.761	0.261	0.964

### 3.2 Feature selection and model performance

#### 3.2.1 Radiomics models

In this study, a total of 1562 HCR features were extracted and their distribution is presented in **Supplementary Figure 2**. After feature selection, 16 HCR features were ultimately chosen for further analysis and construction of traditional radiomics models (**Supplementary Figure 3**). The predictive performance of different classifiers combined is summarized in **Table 4**. Among these models, the ExtraTrees model demonstrated superior predictive performance in the test set, achieving an AUC of 0.853 (95% CI: 0.770 - 0.936). The ROC curve can be found in **Supplementary Figure 4**.

**Table 4:** Performance comparison of different radiomics models.

Model	AUC	95% CI	Accuracy	Sensitivity	Specificity	PPV	NPV
LR-training	0.768	0.711 - 0.826	0.685	0.759	0.663	0.396	0.904
SVM-training	0.946	0.912 - 0.979	0.932	0.892	0.944	0.822	0.968
RandomForest-training	0.864	0.818 - 0.911	0.807	0.771	0.818	0.552	0.925
ExtraTrees-training	0.910	0.874 - 0.946	0.840	0.855	0.835	0.602	0.952
XGBoost-training	0.849	0.799 - 0.899	0.815	0.699	0.849	0.574	0.906
LightGBM-training	0.929	0.897 - 0.960	0.818	0.904	0.793	0.560	0.966
MLP-training	0.960	0.940 - 0.980	0.910	0.904	0.912	0.750	0.970
LR-testing	0.793	0.689 - 0.897	0.722	0.750	0.718	0.231	0.962
SVM-testing	0.754	0.643 - 0.864	0.576	0.812	0.549	0.169	0.963
RandomForest-testing	0.838	0.736 - 0.939	0.829	0.750	0.838	0.343	0.967
ExtraTrees-testing	0.853	0.770 - 0.936	0.848	0.625	0.873	0.357	0.954
XGBoost-testing	0.765	0.656 - 0.874	0.627	0.750	0.613	0.179	0.956
LightGBM-testing	0.778	0.669 - 0.886	0.703	0.687	0.704	0.208	0.952
MLP-testing	0.738	0.606 - 0.870	0.747	0.687	0.754	0.239	0.955

#### 3.2.2 DL models

The performance of three DL models is presented in **Table 5** and **Supplementary Figure 5**. The Densenet121 model demonstrated superior performance compared to the ExtraTrees model based on clinical and traditional radiomics, achieving an AUC of 0.883 (95% CI: 0.817 - 0.947) in the testing set.

**Table 5:** Performance comparison of DL models.

Model.	AUC	95% CI	Accuracy	Sensitivity	Specificity	PPV	NPV
Densenet121-training	0.902	0.866 - 0.938	0.774	0.867	0.747	0.500	0.951
VGG19-training	0.861	0.816 - 0.905	0.802	0.783	0.807	0.542	0.927
Resnet50-training	0.906	0.868 - 0.944	0.883	0.747	0.923	0.738	0.926
Densenet121-testing	0.883	0.817 - 0.947	0.886	0.687	0.908	0.458	0.963
VGG19-testing	0.815	0.694 - 0.935	0.816	0.625	0.838	0.303	0.952
Resnet50-testing	0.793	0.646 - 0.939	0.892	0.562	0.930	0.474	0.950

**Figure 3:**
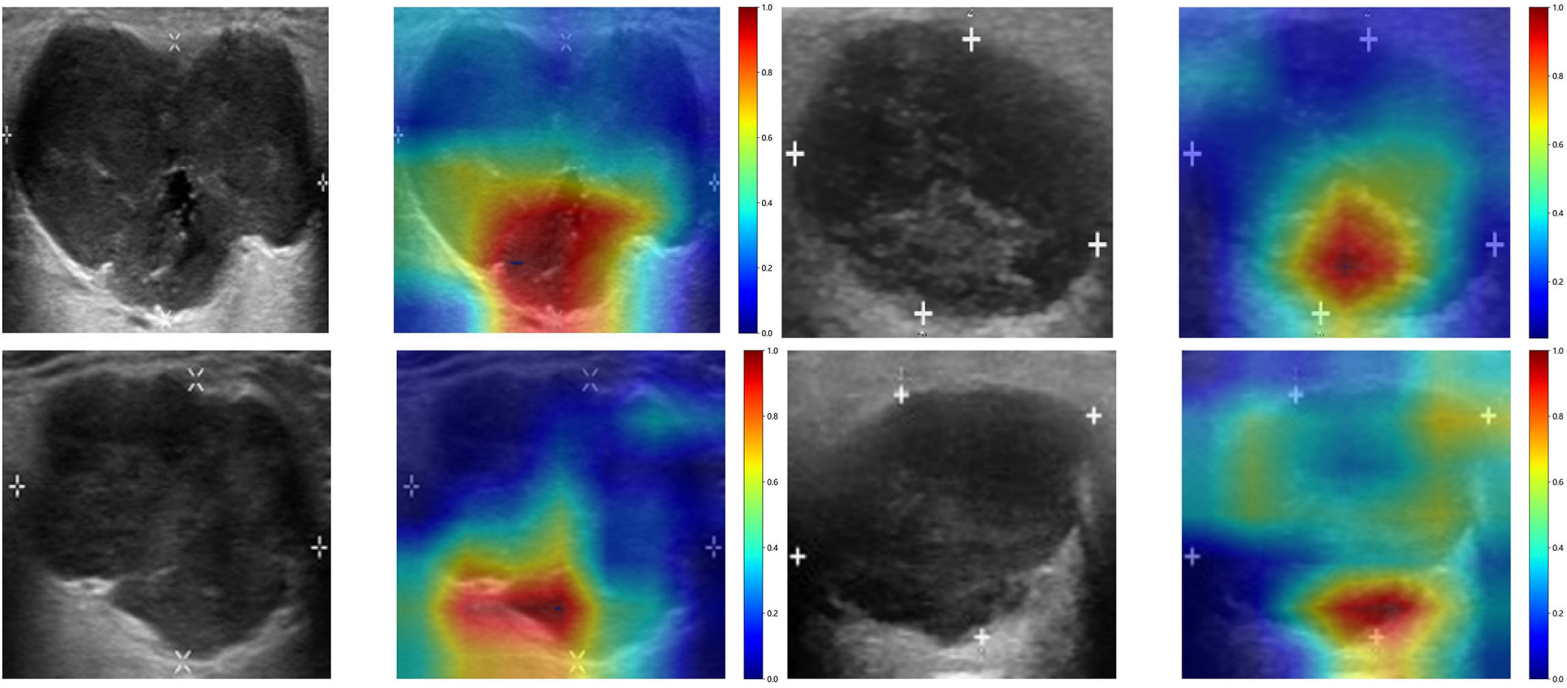
The Grad-CAM visualizations for four typical samples. These visualizations are instrumental in demonstrating how the model focuses on different regions of the images for making its predictions.

To investigate the recognition ability of the Densenet121 model across different samples, we utilized the Gradient-weighted Class Activation Mapping (Grad-CAM) technique for visualization. **Figure 3** demonstrates the application of Grad-CAM, effectively highlighting the activation status of the final convolutional layer relevant to cancer type prediction. This approach facilitates identification of image regions that significantly influence model decisions and provides valuable insights into the interpretability.

#### 3.2.3 Feature fusion models

**Figure 4:**
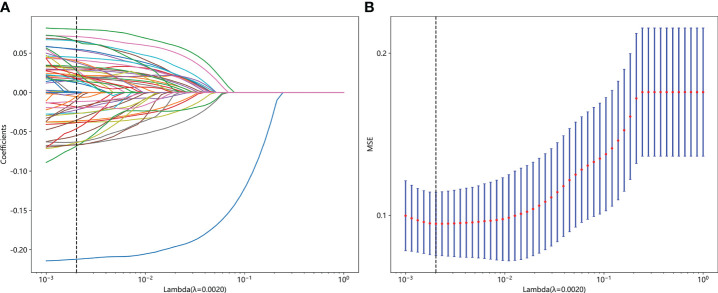
Fusion feature selection using LASSO **(A)** and the histogram of the feature importance score **(B)** based on the selected features. The optimal λ value of 0.0020 was selected.

**Figure 5:**
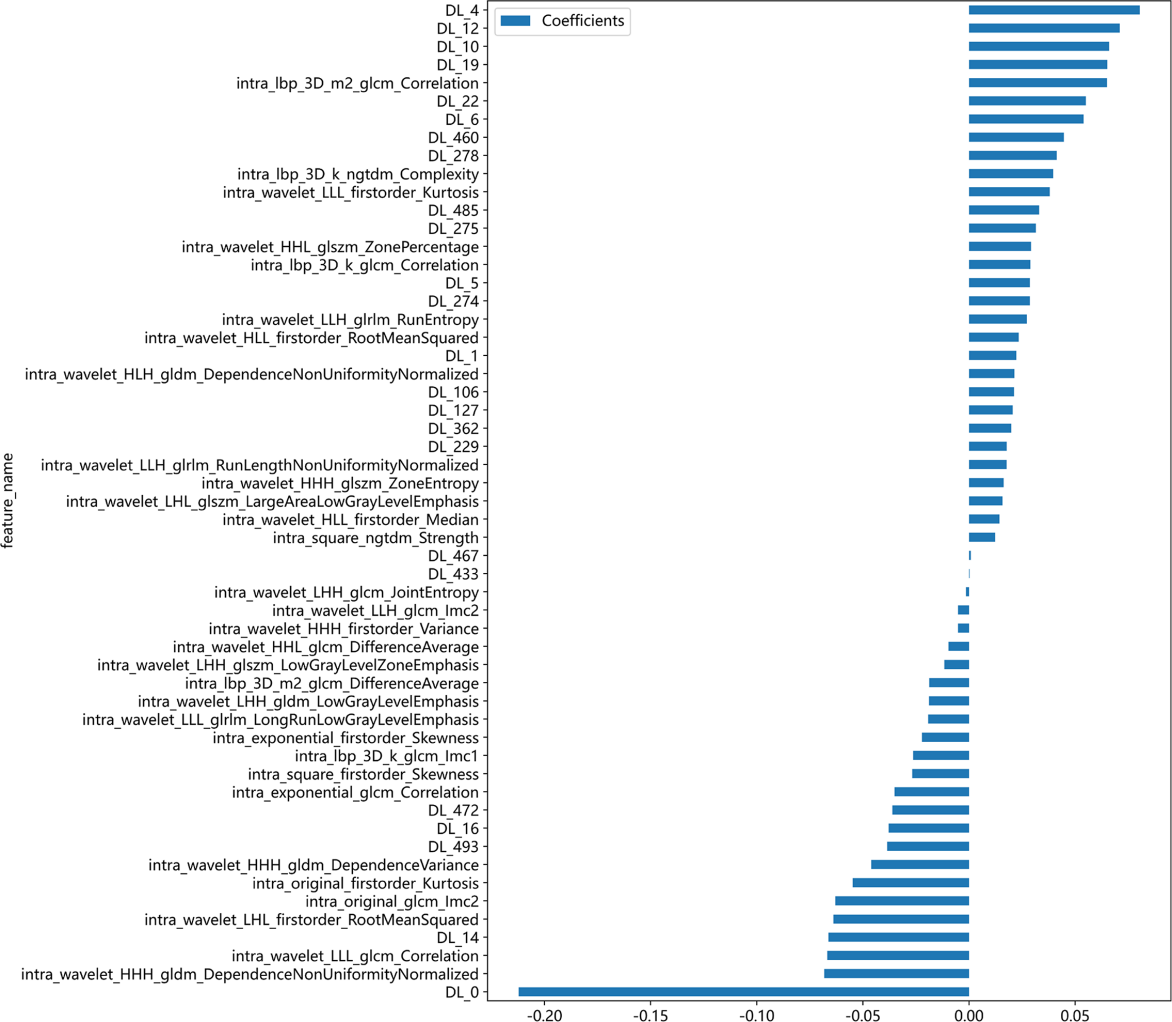
The selected fusion features and corresponding coefficients.

After feature selection, a total of 31 HCR features and 24 DL features were retained from the fused feature set comprising 2,074 dimensions (**Figures 4**, **5**). Subsequently, DLR feature fusion models were constructed by combining multiple classifiers, and the performance comparison is presented in **Table 6** and **Supplementary Figure 6**. The ExtraTrees model achieved an AUC of 0.916 (95% CI: 0.861 - 0.971) in the testing set, demonstrating further enhancement compared to the Densenet121 model (AUC=0.916 vs 0.891).

**Table 6:** Performance comparison of different DLR models.

Model	AUC	95% CI	Accuracy	Sensitivity	Specificity	PPV	NPV
LR-training	0.990	0.982 - 0.997	0.938	0.964	0.930	0.800	0.989
SVM-training	0.998	0.995 - 1.000	0.976	0.988	0.972	0.911	0.996
RandomForest-training	0.971	0.949 - 0.992	0.905	0.940	0.895	0.722	0.981
ExtraTrees-training	0.943	0.918 - 0.969	0.908	0.795	0.940	0.795	0.940
XGBoost-training	0.935	0.908 - 0.962	0.818	0.904	0.793	0.560	0.966
LightGBM-training	0.941	0.917 - 0.965	0.870	0.819	0.884	0.673	0.944
MLP-training	0.989	0.982 - 0.997	0.948	0.976	0.940	0.827	0.993
LR-testing	0.814	0.683 - 0.945	0.880	0.625	0.908	0.435	0.956
SVM-testing	0.831	0.727 - 0.934	0.747	0.750	0.746	0.250	0.964
RandomForest-testing	0.836	0.738 - 0.934	0.665	0.875	0.641	0.215	0.978
ExtraTrees-testing	0.916	0.861 - 0.971	0.892	0.750	0.908	0.480	0.970
XGBoost-testing	0.885	0.815 - 0.955	0.747	0.875	0.732	0.269	0.981
LightGBM-testing	0.870	0.798 - 0.942	0.797	0.812	0.796	0.310	0.974
MLP-testing	0.730	0.571 - 0.890	0.816	0.562	0.845	0.290	0.945

### 3.3 Construction of nomogram and comparison of all models

**Figure 6:**
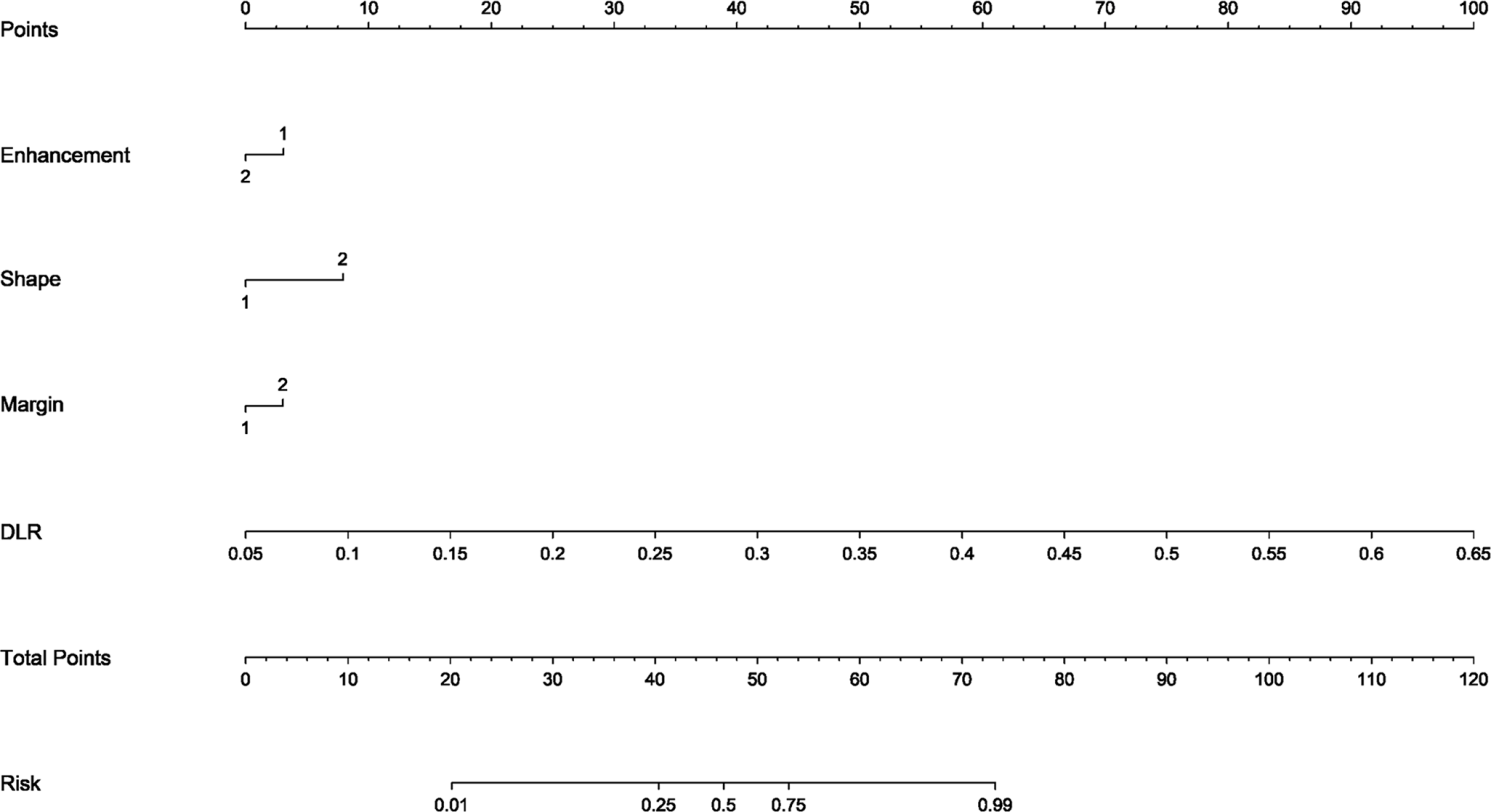
The DLR nomogram for predicting MPGTs. For clinical features in nomogram, 1 means ‘absent’, ’regular’, ’well-defined’, and 2 means ‘present’, ’irregular’, ’poorly-defined’ successively.

The DLR model demonstrated superior performance compared to alternative models, thereby we integrated meaningful clinical features with the DLR model’s predictions for constructing the final combined model, which was effectively visualized by nomogram (DLRN). Nomogram illustrated that DLR factor played a significant role in predicting the risk level of PGTs (**Figure 6**).

**Figure 7:**
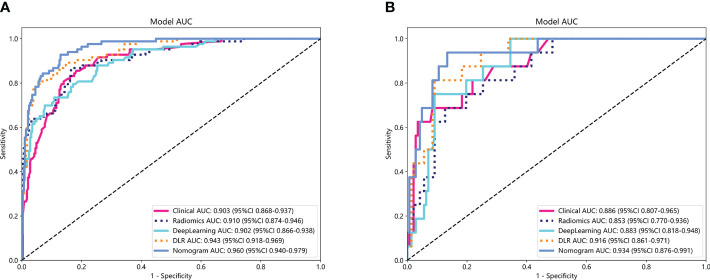
The ROC curves for different models in training set **(A)** and testing set **(B)**. AUC, area under the curves.

**Figure 8:**
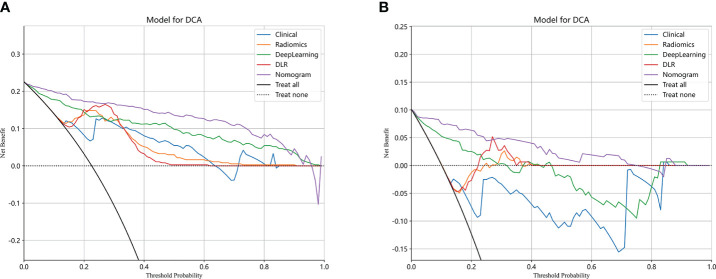
Different models’ DCA curves in training set **(A)** and testing set **(B)**.

The performance of the clinical model, radiomics model, DL model, DLR model, and DLRN was summarized in **Table 7**. Among all models evaluated (**Figure 7**), DLRN exhibited superior performance with an AUC of 0.960 (95% CI: 0.940 - 0.979) for the training set and 0.934 (95% CI: 0.876 - 0.991) for the testing set. Delong test (**Supplementary Figure 7**) revealed statistically significant differences between DLR and DLRN model compared to others in the training set (P < 0.05). However, no statistically significant difference was observed among all models in the testing set (P > 0.05). The calibration curves ((**Supplementary Figure 8**) demonstrated excellent fit for DLRN with a HL test statistic of 0.327 for the training set and 0.793 for the testing set. Furthermore, based on DCA curves results (**Figure 8**), it could be concluded that DLRN provided superior clinical benefits compared to other models.

**Table 7:** Performance comparison of different models.

Model	AUC	95% CI	Accuracy	Sensitivity	Specificity	PPV	NPV
Clinical-training	0.903	0.868 - 0.937	0.826	0.843	0.821	0.579	0.947
Radiomics-training	0.910	0.874 - 0.946	0.840	0.855	0.835	0.602	0.952
Deep Learning -training	0.902	0.866 - 0.938	0.774	0.867	0.747	0.500	0.951
DLR-training	0.943	0.918 - 0.969	0.908	0.795	0.940	0.795	0.940
Nomogram -training	0.960	0.940 - 0.979	0.880	0.916	0.870	0.673	0.973
Clinical-testing	0.886	0.807 - 0.965	0.892	0.625	0.923	0.476	0.956
Radiomics-testing	0.853	0.770 - 0.936	0.848	0.625	0.873	0.357	0.954
Deep Learning -testing	0.883	0.818 - 0.947	0.886	0.687	0.908	0.458	0.963
DLR-testing	0.916	0.861 - 0.971	0.892	0.750	0.908	0.480	0.970
Nomogram -testing	0.934	0.876 - 0.991	0.867	0.875	0.866	0.424	0.984

## 4 Discussion

Our research findings demonstrated that DL models outperformed traditional radiomics models in the classification of PGTs based on US images (AUC=0.883 *vs* 0.853). Furthermore, the performance of feature fusion DLR model further enhanced (AUC=0.916). Clinical and US characteristics also provide valuable information for model construction, and the DLRN model that integrated all available data demonstrated superior performance (AUC=0.934). The DCA curve illustrated that the adoption of DLRN would yield enhanced benefits for patients.

Controversy surrounds the diagnostic value of clinical data and US characteristics for PGTs. BPGTs typically exhibit well-defined margin, homogeneous echogenicity, and posterior acoustic enhancement in US images. In contrast, high-grade malignant tumors often display heterogeneous echogenicity, poorly-defined margin, and internal calcifications (27). However, PGTs encompass a wide range of histological types with diverse cellular origins or differentiations. Additionally, tumor cells can undergo various forms of metaplasia, resulting in variations or overlaps in the pathological and corresponding radiological manifestations. In this study, through univariate and multivariate logistic regression analysis, irregular shape, poorly-defined margin, and absence of posterior acoustic enhancement were identified as independent factors for MPGTs. These findings were consistent with previous studies. The clinical models demonstrated excellent performance in both the training set and testing set (AUC=0.897~0.952, 0.843~0.886).

Radiomics is the process that converts digital medical images into high-dimensional, mineable data. Numerous domestic and international studies have investigated its application in distinguishing PGTs (18–21). Qi et al. (19) conducted a study to differentiate between BPGTs and MPGTs, as well as different subtypes of benign tumors. The results demonstrated that the multi-sequence radiomics model based on conventional MRI exhibited excellent performance in classifying BPGTs and MPGTs, with further improvement when combined with clinical features (AUC=0.863). Li et al. (21) validated the effectiveness of radiomics analysis using conventional ultrasound (CUS) images for preoperative prediction of the malignant potential of parotid lesions. By combining radiomic features, CUS features, and clinical information in the nomogram, the ability to differentiate between benign and malignant parotid lesions was enhanced (AUC=0.91). The traditional radiomics models, combined with diverse classifiers, showed satisfactory diagnostic performance in our study. The training set had an AUC ranging from 0.768 to 0.960, while the testing set ranged from 0.738 to 0.853.

Feature extraction plays a crucial role in radiomics, but conventional radiomics often generate numerous low-level and predefined features that may not fully capture the heterogeneity of images. This limitation restricts the potential of radiomics models. In recent years, the integration of DL and radiomics has gained momentum due to the unique advantages of DL in computer vision and image recognition tasks. DL networks autonomously learn high-level features specific to research problems, enabling a more comprehensive reflection of information within lesions. However, their performance heavily relies on data volume and entails significant computational costs. Transfer learning can be leveraged by utilizing pre-trained DL networks from large-scale datasets like ImageNet and fine-tuning them for extracting DL features from smaller datasets for radiomics analysis. This approach helps mitigate overfitting issues caused by limited data availability and opens up new avenues for advancing radiomics (28). Existing studies have demonstrated that models combining DL features with radiomics features outperform those using either features alone in various clinical problems such as breast tumors (29), renal cystic lesions (30), meningiomas (31),and tuberculosis (32). In our study, while each individual model demonstrated satisfactory performance in isolation, the integration of DL with clinical and radiomics data yielded a more robust predictive tool, effectively capitalizing on the unique strengths of each individual component.

In a recent study examining the application of deep learning in parotid gland tumors, Liu et al. (33) evaluated five DL models (ResNet50, MobileNetV2, InceptionV1, DenseNet121 and VGG16) based on US images to differentiate PA and WT. DL models are superior to ultrasound and FNAC, the AUC value of these DL models in the test set was from 0.828 to 0.908 and ResNet50 demonstrated the optimal performance. In our study, we attempted to utilize various CNNs including Densenet121, VGG19, and Resnet50. The disparities in performance among different DL models can be attributed to variations in their internal network architectures. Specifically, Densenet121 (34) utilizes a dense connection structure wherein the output of each layer is directly connected to the input of all subsequent layers. This architectural design enhances scalability and parameter efficiency while mitigating gradient vanishing issues and expediting model training processes. Visualization using Grad-CAM demonstrated that model decision-making focused on edge areas of tumors predominantly, which aligned with clinical factors and contributed to interpretability of the models.

Selecting an appropriate and efficient modeling classifier is crucial for developing robust models. In the discrimination of BPGTs and MPGTs, Yu et al. (35) utilized SVM and LR paired with three feature selection methods, to construct distinct radiomics models based on multi-phase CT images. The results demonstrated that the SVM model utilizing a combination of three phases exhibited superior predictive performance, achieving an AUC of 0.936 in the testing set. Lu et al. (20) conducted radiomics analysis of PGTs employing five common machine learning classifiers based on plain CT images and observed variations in optimal classification efficacy among different subtypes of PGTs across these classifiers. Notably, the RandomForest model achieved the highest AUC (0.834) in distinguishing between BPGTs and MPGTs, indicating that model performance may be influenced by key tumor features as well as algorithmic characteristics inherent to each classifier. The ExtraTrees classifier demonstrated superior performance in the testing set of clinical, radiomics, and DLR models in our study. By incorporating additional randomness derived from RandomForest, the ExtraTrees effectively reduces model variance and enhances generalization capabilities, making it highly efficient for handling extensive datasets (36).

The rapid advancement of deep learning in computer vision has led to the emergence of highly competitive approaches in tumor-related domains through the integration of multi-modal and multi-omics features. Zhang et al. (37) proposed two multi-sequence networks (ResFN-Net and FN-Net),based on ResNet and ConvNeXt network respectively, incorporating attention mechanism for the classification of CDKN2A/B homozygous deletion status in IDH-mutant astrocytomas using CE-T1WI and T2WI MRI images. The FN-Net deep learning network based on ConvNeXt demonstrated superior predictive performance with an ACC of 0.9236 and an AUC of 0.9704. ConvNeXt network builds upon ResNet, drawing inspiration from the Swin Transformer and incorporating Spatial Pyramid Pooling (SPP) technology to effectively capture intricate details and global features. It has demonstrated comparable accuracy, scalability, and robustness as Transformer (38). The study conducted by Vanguri et al. (39) published in “Nature cancer” showcased the value of multimodal integration as well. Researchers have developed a multimodal DyAM model that combines histology, radiology, and genomics to accurately predict immunotherapy response in NSCLC patients. The model (AUC = 0.80, 95% CI 0.74-0.86) outperformed unimodal measures, including tumor mutation burden and programmed deathligand-1 immunohistochemistry score. These findings suggest that machine learning techniques combining multiple modalities have complementary and synergistic effects, facilitating oncology decision-making.

The present study is subject to certain limitations. Firstly, the retrospective design employed in this study may introduce potential selection bias. Secondly, patients were recruited from a single-center medical institution and lacked external validation. Future research should involve multi-center participation to expand the sample size and enhance model generalizability. Lastly, our feature extraction and model construction solely relied on conventional two-dimensional US images with manually delineated ROI, without incorporating other modalities such as elastography or contrast-enhanced imaging. Utilizing standardized single-modality images allows for easier acquisition and wider applicability and dissemination of the model. In future studies, we will concentrate on constructing models using multi-modal imaging to extract comprehensive information and integrating deep learning automatic segmentation algorithms to improve delineation accuracy and repeatability, thereby enhancing diagnostic performance.

## 5 Conclusions

This study demonstrated that the feature fusion DLR model based on US images exhibit superior classification performance in distinguishing between BPGTs and MPGTs, compared to clinical models, traditional radiomics models, and DL models. Moreover, by incorporating clinical and US factors, the performance of DLRN is further enhanced. This model holds immense potential for facilitating individualized diagnosis and treatment plans in clinical settings, thereby contributing to precision medicine.

## Data availability statement

The raw data supporting the conclusions of this article will be made available by the authors, without undue reservation.

## Ethics statement

The studies involving humans were approved by The Ethics Committee of The Fourth Hospital of Hebei Medical University. The studies were conducted in accordance with the local legislation and institutional requirements. Written informed consent for participation was not required from the participants or the participants’ legal guardians/next of kin in accordance with the national legislation and institutional requirements.

## Author contributions

YiW: Conceptualization, Data curation, Investigation, Writing – original draft. JG: Investigation, Writing – original draft. ZY: Resources, Writing – original draft. YuW: Resources, Writing – review & editing. MS: Data curation, Writing – review & editing. RH: Writing – review & editing.
