# Supplementary material for: Differentiation of benign and malignant parotid gland tumors based on the fusion of radiomics and deep learning features on ultrasound images
Source: Front Oncol. 2024 May 13;14:1384105. doi: 10.3389/fonc.2024.1384105 (PMC11128676; doi:10.3389/fonc.2024.1384105)
Supplement: Supplementary file 1 [file DataSheet_1.docx]

Supplementary Material

**1.**The cosine decay learning rate strategy in deep transfer learning defined as follows:

$$\eta_{t}=\eta_{min}^{i}+\frac{1}{2}\left( \eta_{max}^{i}-\eta_{min}^{i} \right)\left( 1+cos\left( \frac{T_{cur}}{T_{i}}\pi\right) \right)$$

The notation$\eta_{min}^{i}=0$sets the minimum learning rate, while $\eta_{max}^{i}=0.01$establishes the maximum learning rate. The term $T_{i}=30$ denotes the number of epochs in the iterative training process. Other essential hyperparameters include the use of Stochastic Gradient Descent (SGD) as the optimizer and softmax cross-entropy for the loss function.

**2. Clinical and US characteristics**

**Supplementary Table 1.** Univariable and multivariable analysis of clinical and US characteristics.

| Clinical and US  characteristics | Univariable Analysis | | | |  | Multivariable Analysis | | | |
| --- | --- | --- | --- | --- | --- | --- | --- | --- | --- |
|  | OR | Lower 95%CI | Upper 95%CI | *P* |  | OR | Lower 95%CI | Upper 95%CI | *P* |
| Posterior acoustic enhancement | 0.681 | 0.630 | 0.736 | <0.05 |  | 0.807 | 0.752 | 0.865 | <0.05 |
| Smoking history | 0.885 | 0.820 | 0.955 | <0.05 |  | 0.943 | 0.888 | 1.002 | 0.114 |
| Drinking history | 0.960 | 0.886 | 1.039 | 0.391 |  |  |  |  |  |
| Echogenicity | 0.974 | 0.890 | 1.066 | 0.632 |  |  |  |  |  |
| Age | 1.000 | 0.998 | 1.002 | 0.908 |  |  |  |  |  |
| Gender | 1.010 | 0.939 | 1.085 | 0.826 |  |  |  |  |  |
| Cystic component | 1.019 | 0.933 | 1.113 | 0.720 |  |  |  |  |  |
| Maximum diameter | 1.046 | 1.008 | 1.084 | <0.05 |  | 1.003 | 0.973 | 1.033 | 0.891 |
| Calcification | 1.193 | 1.080 | 1.317 | <0.05 |  | 1.048 | 0.968 | 1.134 | 0.333 |
| Shape | 1.635 | 1.531 | 1.745 | <0.05 |  | 1.257 | 1.155 | 1.368 | <0.05 |
| Margin | 1.669 | 1.565 | 1.779 | <0.05 |  | 1.323 | 1.215 | 1.439 | <0.05 |

**3. Clinical models**


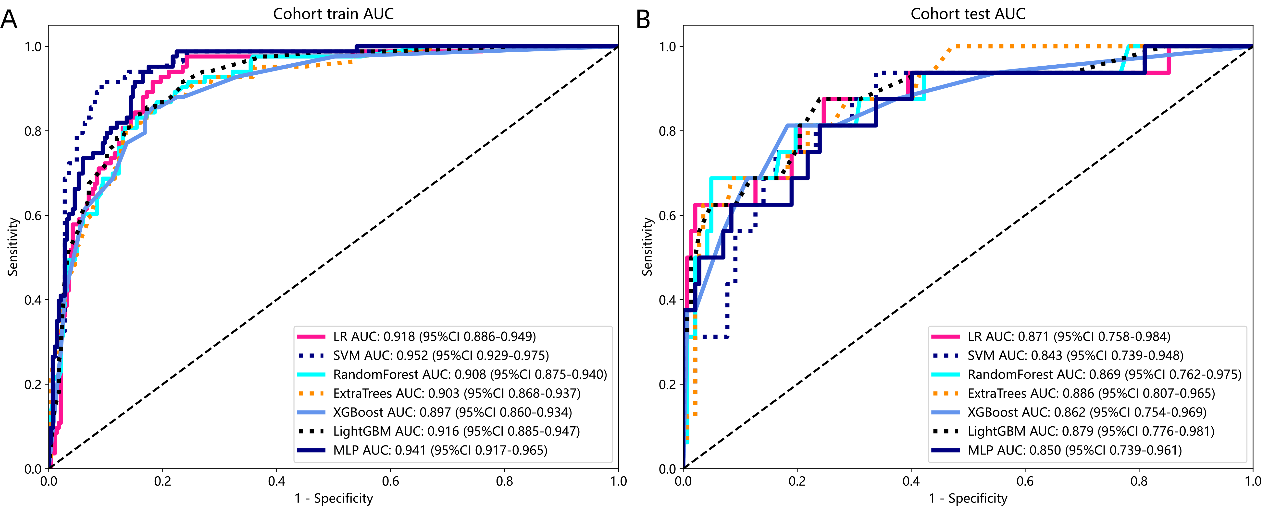


**Supplementary Figure 1.** ROC curves for clinical models in training set (**A**) and testing set (**B**).

**4. Radiomics models**


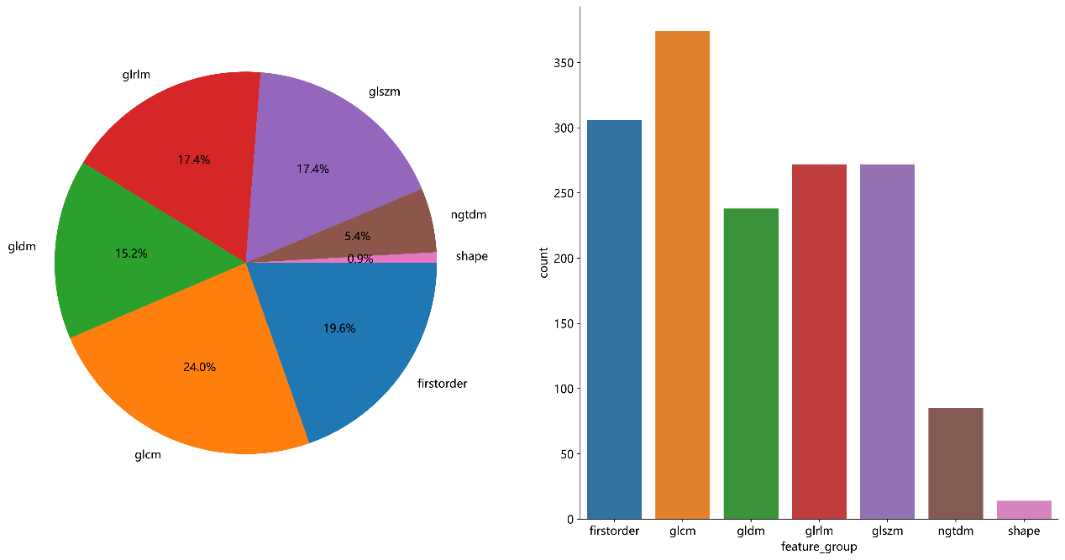


**Supplementary Figure 2.**  Number and ratio of handcrafted radiomics features.


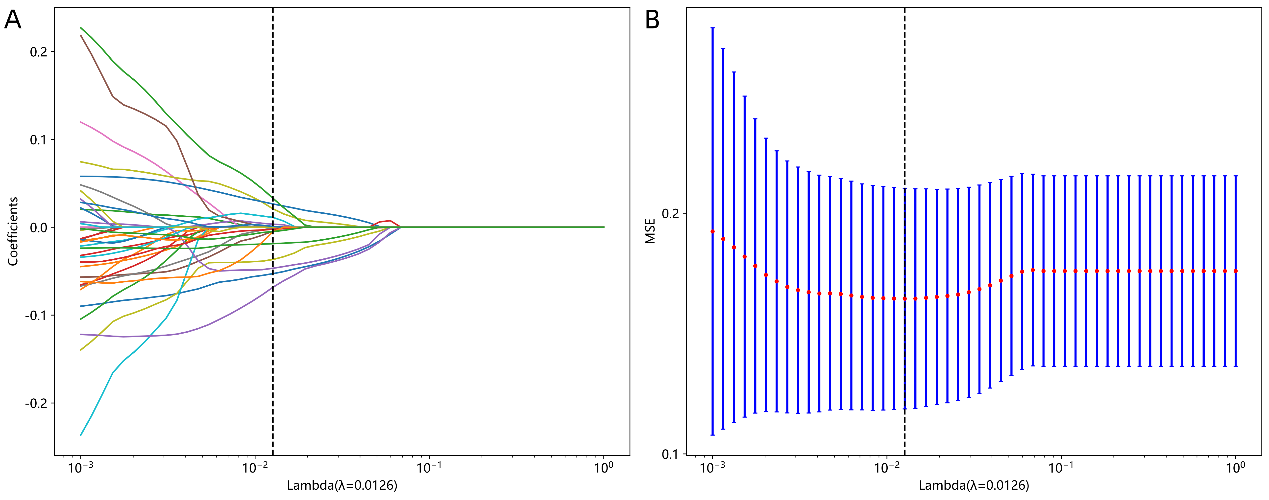


**Supplementary Figure 3.** Handcrafted radiomics feature selection using LASSO (**A**)and the histogram of the radiomics feature importance score(**B**) based on the selected features. The optimal λ value of 0.0126 was selected.


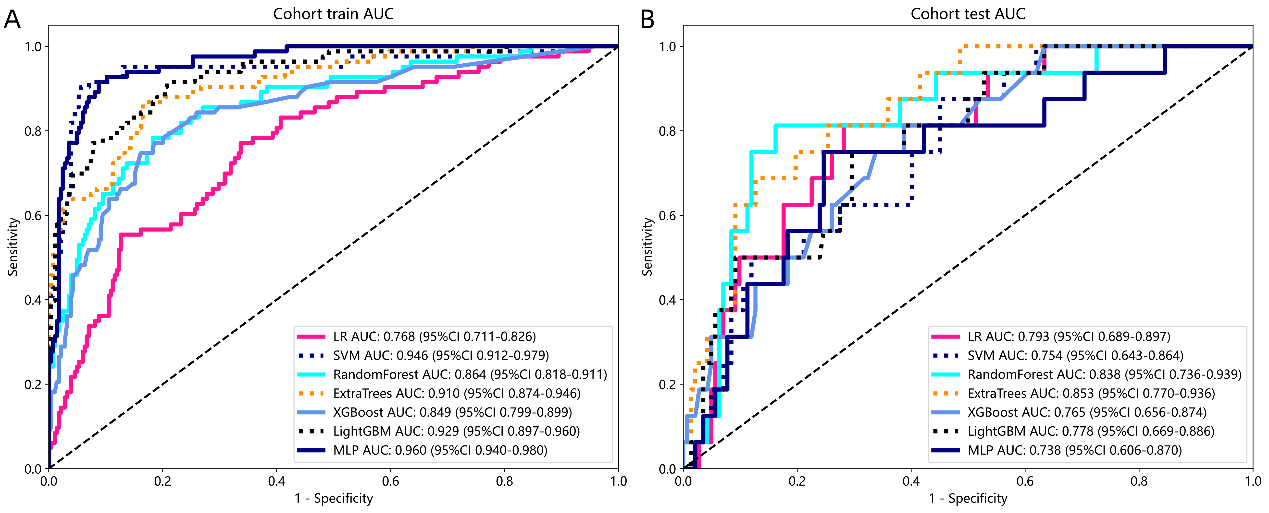


**Supplementary Figure 4.** ROC curves for radiomics models in training set (**A**) and testing set (**B**).

**5. Deep learning (DL) models**

**
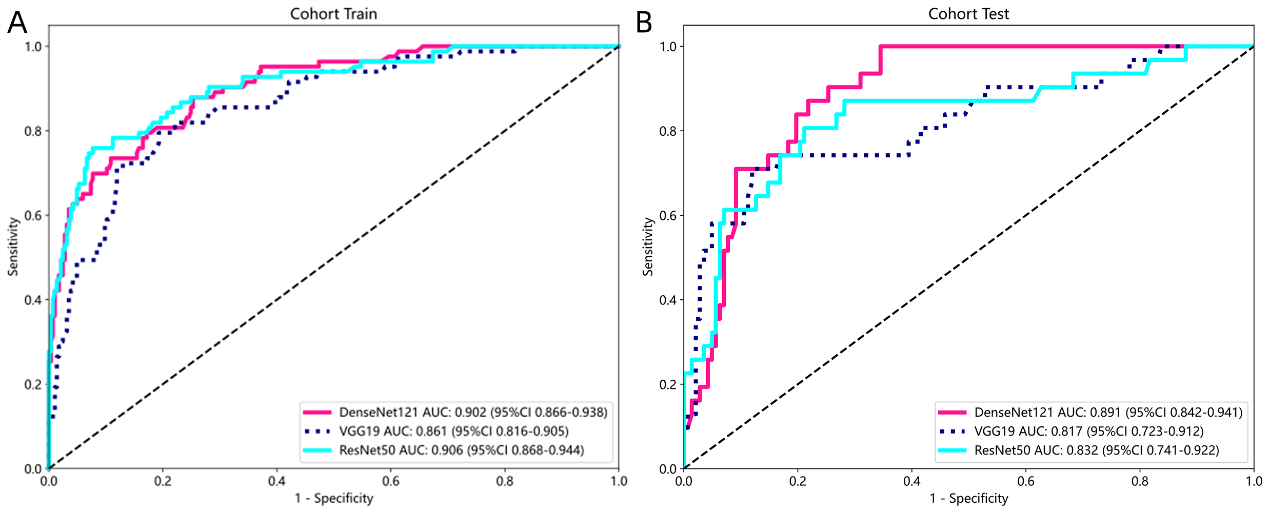
**

**Supplementary Figure 5.** ROC curves for DL models in training set (**A**) and testing set (**B**).

**6.Deep learning radiomics (DLR) models**

**
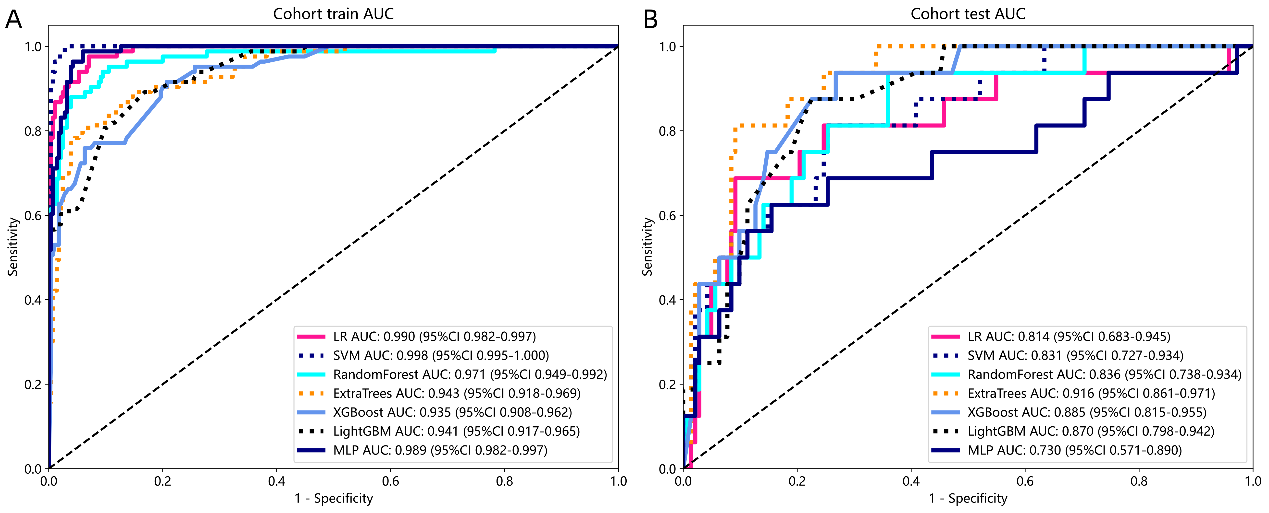
**

**Supplementary Figure 6.** ROC curves for DLR models in training set (**A**) and testing set (**B**).

**7.** **Delong Test**

**
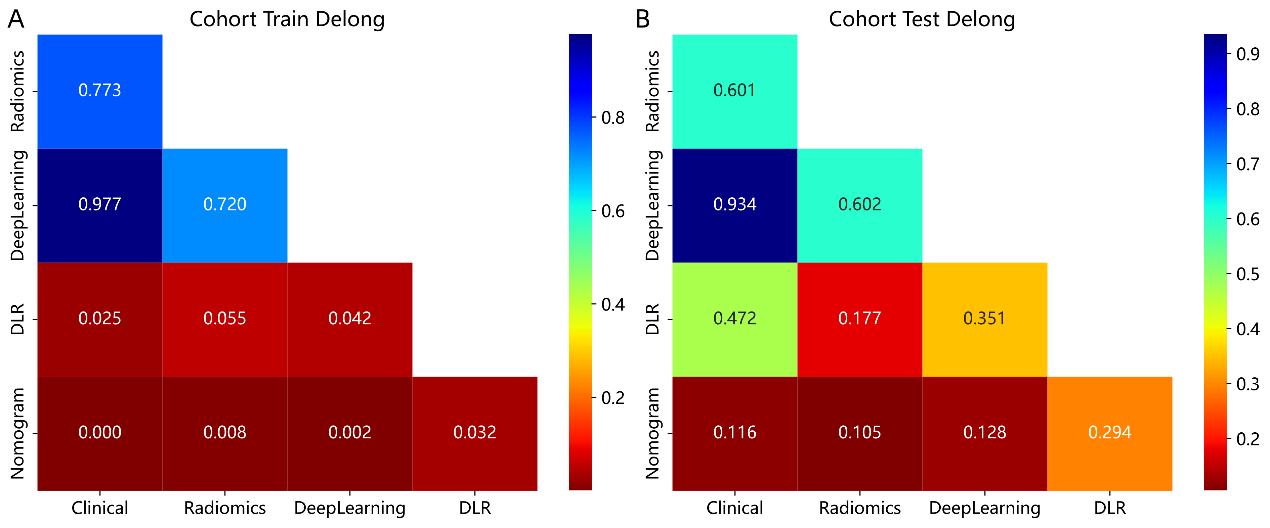
**

**Supplementary Figure 7.** Delong test in training set (**A**) and testing set (**B**).

**8. Calibration curves**

**
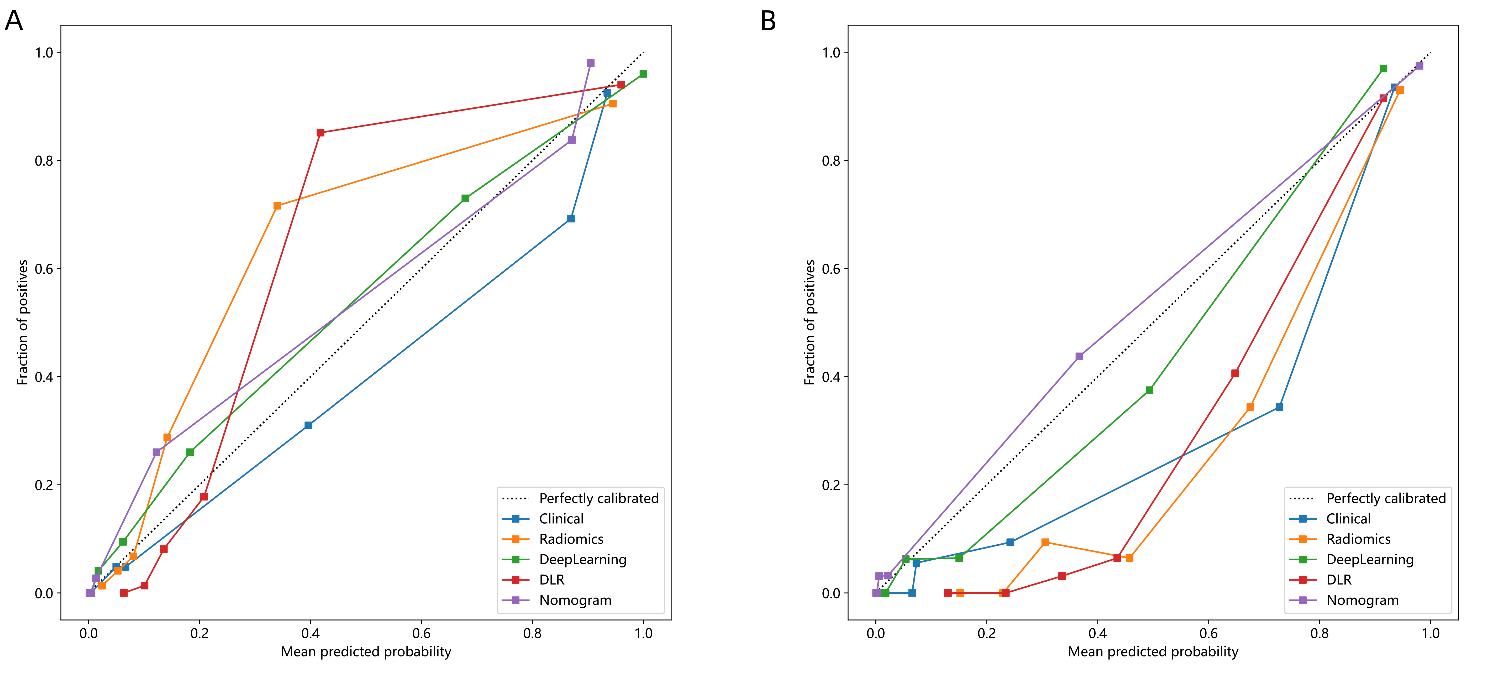
**

**Supplementary Figure** **8.** The calibration curves for different models in training set (**A**) and testing set (**B**).
